# Influence of two different surgical techniques on the difficulty of impacted lower third molar extraction and their post-operative complications

**DOI:** 10.4317/medoral.20605

**Published:** 2015-06-27

**Authors:** Alexandra Mavrodi, Ani Ohanyan, Nikos Kechagias, Antonis Tsekos, Konstantinos Vahtsevanos

**Affiliations:** 1 DDS, Department of Oral & Maxillofacial Surgery Clinic, Aristotle University of Thessaloniki, Thessaloniki, Greece; 2 MD, DDS, OMFS, PhD, Division of Oral & Maxillofacial Surgery, Clinic St. Loukas, Thessaloniki, Greece; 3 MD, DDS, OMFS, Oral & Maxillofacial Department, Army General Hospital of Thessaloniki, Thessaloniki, Greece; 4 MD, DDS, OMFS, PhD, Assist. Prof., Department of Oral & Maxillofacial Surgery Clinic, Aristotle University of Thessaloniki, Thessaloniki, Greece

## Abstract

**Background:**

Post-operative complications of various degrees of severity are commonly observed in third molar impaction surgery. For this reason, a surgical procedure that decreases the trauma of bone and soft tissues should be a priority for surgeons. In the present study, we compare the efficacy and the post-operative complications of patients to whom two different surgical techniques were applied for impacted lower third molar extraction.

**Material and Methods:**

Patients of the first group underwent the classical bur technique, while patients of the second group underwent another technique, in which an elevator was placed on the buccal surface of the impacted molar in order to luxate the alveolar socket more easily.

**Results:**

Comparing the two techniques, we observed a statistically significant decrease in the duration of the procedure and in the need for tooth sectioning when applying the second surgical technique, while the post-operative complications were similar in the two groups. We also found a statistically significant lower incidence of lingual nerve lesions and only a slightly higher frequency of sharp mandibular bone irregularities in the second group, which however was not statistically significant.

**Conclusions:**

The results of our study indicate that the surgical technique using an elevator on the buccal surface of the tooth seems to be a reliable method to extract impacted third molars safely, easily, quickly and with the minimum trauma to the surrounding tissues.

**Key words:**Mandibular third molar, impacted, surgical technique, extraction, elevator.

## Introduction

Owing to the fact that at least one impacted third molar can be traced in 33% of the general population (1,2), impacted third molars, especially mandibular ones, constitute a common cause of pain and inflammation in the oral region. The high prevalence of their impaction has been attributed to a remarkable variety of factors, among which are inadequate retromolar space, unfavorable path of eruption, malposition of the tooth germ and hereditary reasons (3). Impacted third molars are associated with numerous complications, such as pericoronitis, periodontal pathology or root resorption of the adjacent tooth, caries, cystic or neoplastic lesions, orthodontic or prosthetic problems and temporomandibular joint symptoms (4,5).

Therefore, third molar impaction surgery is a common procedure, with multiple, however, postoperative complications, which are not only a cause for patient’s discomfort but a source of surgeon’s concern as well. Specifically, the majority of patients who undergo a surgical extraction of an impacted third molar suffer from pain, swelling, trismus and general oral discomfort during the first postoperative days. On the other hand, less frequent complications might be alveolitis, infection, hemorrhage and nerve injury (6). The severity of the aforementioned symptoms depends on a series of factors including the experience of the surgeon, the duration and the difficulty of surgery, the extent of bone removal and the level of oral hygiene (7). Consequently, it is of major importance that the surgeon chooses a surgical technique that renders the extraction of the impacted tooth as atraumatic as possible.

In the present study, we compare the efficacy of two surgical techniques and their postoperative complications after impacted lower third molar extraction.

## Material and Methods

The current study was performed on compliance with the Principles of the Helsinki Declaration and was given institutional ethical approval. Additionally, all patients received prior to the intervention a document that described the procedure and signed an informed consent.

Each patient seeking medical treatment which involved surgical extraction of an impacted lower third molar was randomly classified into one of the two groups designed for the study. As a consequence patients of all ages and both sexes and impacted teeth of all degrees of surgical difficulty were included in both of the groups described as follows. All patients fulfilled the following inclusion criteria: they presented with an impacted lower third molar and underwent a surgical extraction irrespective of the reason why this was recommended. Exclusion criteria were: a) patients presenting with pregnancy or significant systemic disease, including advanced carcinoma, autoimmune diseases or associated bone pathology, b) patients undergone or undergoing chemotherapy or radiotherapy and c) immunocompromised patients.

Data collected was analyzed by SPSS software version 22 for Windows using the independent samples t-test and the Chi-Square test at a significance level of *P*<0.05.

From September 2011 to July 2014 we removed surgically 1210 impacted lower third molars, fulfilling the aforementioned inclusion criteria Regarding the gender, 57.9% of the patients treated were female and 42.1% male. The age of female patients ranged from 15 to 78 years with an average of 46.5 years, whereas the age of male patients varied between 16 and 82 years with a mean of 49 years. The average age of all patients, both male and female, was 48.5 years. As for the side of the operation, 47.3% of the extracted third molars were left, while 52.7% were right. All patients were randomly treated by three surgeons.

We extracted 470 of the aforementioned third molars (first group) with the classical surgical bur technique. All surgeries of this first group were performed under local anesthesia. Approximately 1.6-2.4 ml of anesthetic solution (2% lidocaine with 1:100.000 epinephrine) was used and in addition to inferior alveolar nerve block, infiltration anesthesia was placed in the area overlying the third molar. A full-thickness mucoperiosteal 3-cornered flap was elevated to allow adequate visualization and placement of retractors, drilling equipment, elevators and forceps with the minimum trauma to the surrounding soft tissues. Depending on the depth of impaction and the topographical relationship of the third molar to the anterior border of the ramus, the surgeon used a hand piece with adequate speed and torque to remove bone from the occlusal, buccal and distal aspect of the impacted molar, whenever he or she considered it as necessary. A straight elevator was then used in the mesial aspect of the third molar in order to expand the alveolar socket and elevate the tooth. Where necessary, the surgeon sectioned the tooth so as to safely extract it from its socket in pieces. After the removal of any follicular, bone or tooth fragments and the irrigation of the alveolar socket with saline, simple interrupted stitch was applied for primary closure of the wound. All patients received antibiotics for 5 days and non steroidal anti-inflammatory drugs for the first 2 postoperative days and the next 2 days, too, if they suffered from pain. The sutures were removed 8 days after the surgery.

**Figure 1:**
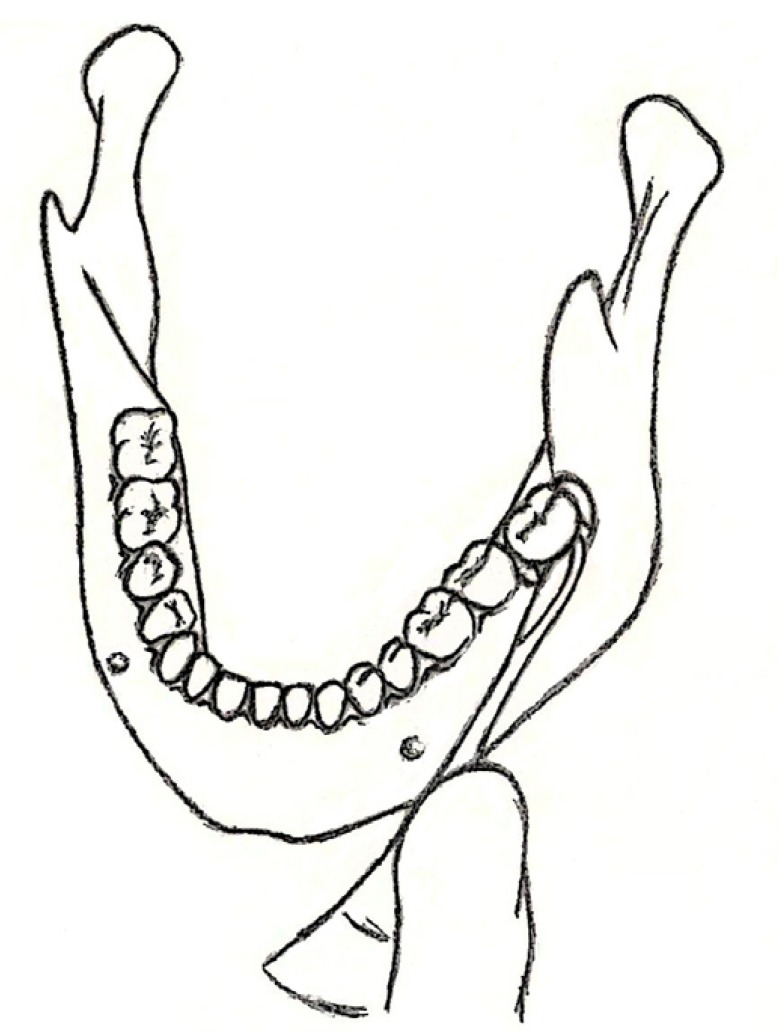
Schematic representation of the application of the straight elevator in the buccal surface of the tooth. Soft tissues are not depicted in order to display in detail the exact location where the elevator is positioned.

**Figure 2:**
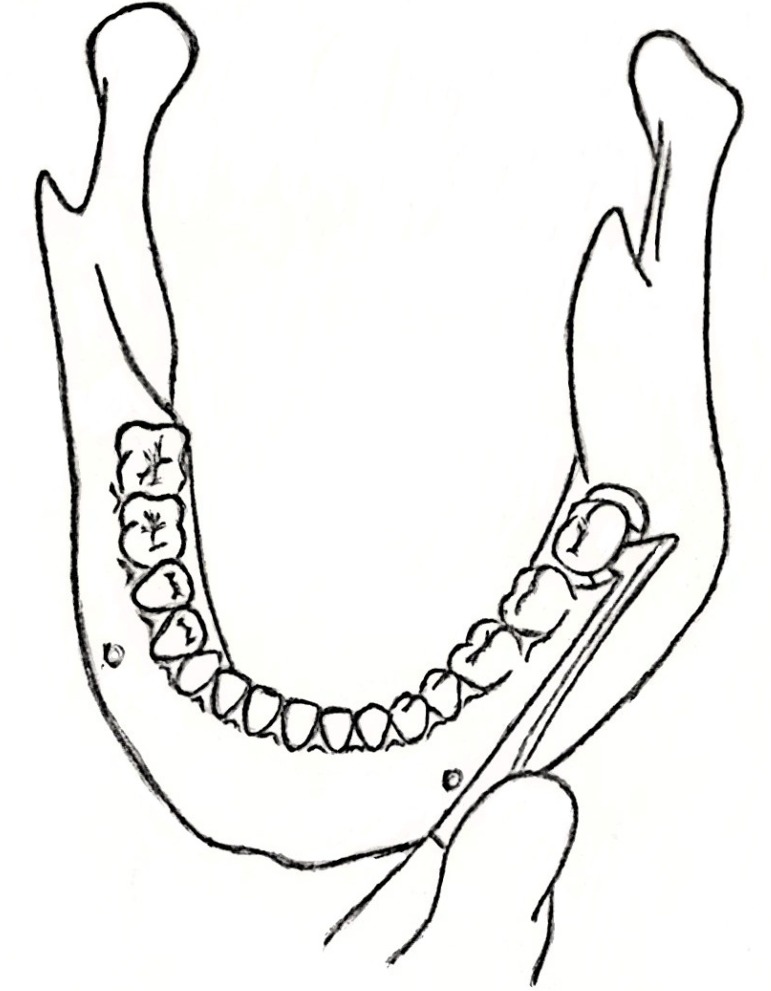
Schematic representation of the cryer elevator applied in the buccal surface of the tooth. Soft tissues are not depicted in order to display with accuracy the exact location where the elevator is positioned.

The rest 740 impacted lower third molars (second group) were removed with a variation of the surgical technique that we have already described. The same anesthetic techniques along with the same 3-cornered flap were used. Nevertheless, after using a straight elevator for the initial luxation of the tooth, the surgeon additionally applied a narrow straight elevator in the mesiobuccal corner of the impacted molar. Gradually and as the alveolar socket expanded, the surgeon was able to reposition the elevator in the buccal surface of the third molar and replace it with a wider one (Fig. 1). The application of the elevator in the buccal surface of the tooth impressively quickly expanded the alveolar socket and as a result the straight elevator could then be replaced by a cryer or a winter elevator (Fig. 2). These latter elevators when applied with a gentle and controllable force elevated easily the impacted tooth and allowed its extraction in a lingual direction. Particularly, when the impacted tooth had two separated roots, the nib of the elevator could be forced under the division of the roots, resulting in an even more quick extraction. After the tooth had been extracted and the alveolar socket examined for any fragments, the same suture technique was applied to this group of patients, who were also given antibiotics and non steroidal anti-inflammatory drugs, too.

## Results

The second third molar extraction technique remarkably decreased the difficulty of the surgeries. Specifically, in comparison to the first group which was treated with the classical surgical bur technique, in the second group less bone was drilled from the contour of the tooth and less frequently tooth sectioning was proved to be necessary. In particular, tooth sectioning was performed in 57.4% of the cases in the first group, and in 32.7% in the second group. Statistical analysis revealed that tooth sectioning was significantly less frequent in the second group (*P*<0.05).

In addition, the surgical procedure was more brief in the second group with an average duration of 22 minutes in comparison to the first group, in which the mean duration was 24.6 minutes. According to statistical analysis the difference is considered significant (*P*<0.05).

The experience of pain and swelling on the operated side was similar for the patients of both groups, who experienced edema that lasted for the first approximately 4 postoperative days, whereas 9 days after the surgery on average the chewing ability of the patients on the side of the extraction returned to normal.

No injury of the lingual nerve and no excessive hemorrhage were recorded in either of the groups while a temporary inferior alveolar nerve paresthesia was reported by 17 patients (3.6%) in the first group and none of the patients of the second group. According to statistical analysis the aforementioned difference is statistically significant (*P*<0.05).

On the other hand, 21 patients of the second group (2.8%) presented with painful sharp mandibular bone irregularities on the lingual cortical plate of the extracted third molar in contrast to 6 patients of the first group (1.3%). The statistical analysis revealed no significant difference between the two groups (*P*<0.05).

## Discussion

The surgical technique that was applied to the second group in the present study was proved to be a safe and efficient method for removing an impacted lower third molar. The lingual cortical plate is thinner than the buccal and as a consequence it can be easily widened allowing the impacted molar to be extracted with a lingual inclination. The application of controllable and not too excessive force and the gradual repositioning of the elevators along the mesiobuccal surface of the tooth in combination with the consecutive replacement of wider elevators obliterate the possibility of a fracture of the lingual plate, which only becomes more elastic. Additionally, the buccal cortical plate, due to the fact that it is supported by the external oblique ridge, is resistant enough to serve as a fulcrum for the elevators.

In this way, despite the fact that an impacted third molar might be too wide mesiodistally to be removed from the alveolar socket when it is luxated by a straight elevator applied in the mesial aspect of the tooth, it can be extracted with a lingual inclination irrespectively of its dimensions. By applying the elevators in the buccal surface of the tooth, the surgeon reduces the need for bone removal and even tooth sectioning, as proven by the present study. Since, the trauma of the surrounding tissues, especially bone tissues, is considerably less with this method, the postoperative complications should also be milder and the recovery faster. However, the present study failed to prove this assumption.

The extraction of the tooth with a lingual inclination raises some concern for the safety of the lingual nerve. Nonetheless, we observed no lingual nerve hypoesthesia or paresthesia among the postoperative complications of the patients treated with the second technique. This could be attributed to the fact that all surgical instruments are applied far away from the route of the lingual nerve which is protected by the lingual cortical plate. In the literature, the frequency of temporary lingual nerve lesions has been reported to range from 0.2 to 23%, while the incidence of permanent lingual nerve injuries varies between 0 to 2% (6,8). Previous studies have shown that increased age, depth of impaction, difficulty or duration of the surgery, lingual flap retraction and tooth sectioning might be risk factors for lingual nerve injuries (6,8). In the second group of the present study not only the duration of the surgery but also the need to section the tooth was decreased. As a consequence, the possibility to injure the lingual nerve was diminished.

The only disadvantage of the second surgical technique seems to be the risk of post-operative sharp mandibular bone irregularities on the lingual plate due to the buccal-lingual elevation. Alves-Pereira *et al*. (9) found sharp mandibular bone irregularities in 0.84% of the cases and reported as risk factors the increased age, the operated side and the presence of radiolucency. They also supported that erupted or nearly erupted third molars might also be associated with more frequent sharp mandibular bone irregularities, since due to their location the lingual plate tends to be thinner and sharper (9). Our study revealed a higher incidence of a sharp alveolar ridge (2.8%), which nonetheless wasn’t statistically significant in relation to the control group. The higher frequency might be associated with the use of winter elevators. These elevators have a T-shaped handle and allow the application of greater force that is difficult to be controlled. However, cryer and straight elevators have proved to be adequately safe as soon as they are applied with controllable force.

Conclusively, the application of the elevators on the buccal surface of the impacted third molar, the lingual elevation of the tooth and its extraction with a lingual inclination is a safe surgical technique. In spite of the slightly higher incidence of postoperative mandibular bone irregularities, which, however, can be prevented by the correct use of surgical instruments, the appropriate application of the elevators on the buccal surface of the impacted molar can impressively reduce the duration of the procedure, the need for excessive bone removal and even tooth sectioning.
